# The Cannabinoid 1 Receptor (*CNR1*) 1359 G/A Polymorphism Modulates Susceptibility to Ulcerative Colitis and the Phenotype in Crohn's Disease

**DOI:** 10.1371/journal.pone.0009453

**Published:** 2010-02-26

**Authors:** Martin Storr, Dominik Emmerdinger, Julia Diegelmann, Simone Pfennig, Thomas Ochsenkühn, Burkhard Göke, Peter Lohse, Stephan Brand

**Affiliations:** 1 Division of Gastroenterology, Department of Medicine, University of Calgary, Calgary, Alberta, Canada; 2 Department of Medicine II – Grosshadern, Ludwig-Maximilians-University Munich, Munich, Germany; 3 Department of Clinical Chemistry – Grosshadern, Ludwig-Maximilians-University Munich, Munich, Germany; Health Canada, Canada

## Abstract

**Background:**

Recent evidence suggests a crucial role of the endocannabinoid system, including the cannabinoid 1 receptor (CNR1), in intestinal inflammation. We therefore investigated the influence of the *CNR1* 1359 G/A (p.Thr453Thr; rs1049353) single nucleotide polymorphism (SNP) on disease susceptibility and phenotype in patients with ulcerative colitis (UC) and Crohn's disease (CD).

**Methods:**

Genomic DNA from 579 phenotypically well-characterized individuals was analyzed for the *CNR1* 1359 G/A SNP. Amongst these were 166 patients with UC, 216 patients with CD, and 197 healthy controls.

**Results:**

Compared to healthy controls, subjects A/A homozygous for the *CNR1* 1359 G/A SNP had a reduced risk to develop UC (p = 0.01, OR 0.30, 95% CI 0.12–0.78). The polymorphism did not modulate CD susceptibility, but carriers of the minor A allele had a lower body mass index than G/G wildtype carriers (p = 0.0005). In addition, homozygous carriers of the G allele were more likely to develop CD before 40 years of age (p = 5.9×10^−7^) than carriers of the A allele.

**Conclusion:**

The CNR*1* p.Thr453Thr polymorphism appears to modulate UC susceptibility and the CD phenotype. The endocannabinoid system may influence the manifestation of inflammatory bowel diseases, suggesting endocannabinoids as potential target for future therapies.

## Introduction

Anecdotal reports suggest that marijuana- or tetrahydrocannabinol-containing products may be effective in alleviating symptoms in patients with ulcerative colitis (UC) and Crohn's disease (CD). [1], [2] This is supported by recent studies of our group and others suggesting that pharmacological activation of the cannabinoid 1 (CB_1_) receptor with selective receptor agonists decreases the inflammatory response in various murine models of colonic inflammation including dinitrobenzene sulphonic acid (DNBS)-, trinitrobenzene sulphonic acid (TNBS)- and dextran sodium sulfate (DSS)-induced colitis. [3]–[7] Interestingly, pharmacological blockade of CB_1_ receptors or genetic ablation of CB_1_ receptors (*CNR1* ^−/−^ mice) aggravates intestinal inflammation in these models, [3], [7] emphasizing the physiological relevance of the CB_1_ receptor in the protection against intestinal inflammation. Increased mucosal levels of the endocannabinoid anandamide during intestinal inflammation in humans further stress the role of the CB_1_ receptor and the endocannabinoid system in intestinal inflammation. [4] Thus, present knowledge suggests up-regulation of endocannabinoids as an important protective mechanism in intestinal inflammation.

The endocannabinoid system and the CB_1_ and CB_2_ receptors seem to be crucially involved in the regulation of multiple physiological functions, e.g. in the heart, where they relax coronary arteries and decrease cardiac work, [8] in organ perfusion, [9] in metabolic homeostasis, [10], [11] and in the regulation of bone mass by osteoclasts, [12] as well as in the protection against stress responses, inflammation, and associated repair mechanisms. [13], [14] Although recent evidence suggests that the endocannabinoid system is involved in many physiological and pathophysiological functions of the gastrointestinal tract such as intestinal motility, secretion, and intestinal inflammation [3], [15]–[20], the exact mechanisms underlying these findings are not yet known. It was recently suggested that CB_1_ signaling may be up-regulated during colitis, [3] but it is unknown whether this is a specific feature of the colitis model or a general response to intestinal inflammation.

Moreover, the role of the CB_1_ receptor in human inflammatory bowel disease (IBD) has not been clarified. Increased anandamide levels were found in mucosal biopsies from UC patients, suggesting a role of the endocannabinoid system in UC. [4] In contrast, the colonic expression of the endocannabinoid 2-acyl-glycerol (2-AG) is not increased in UC. [4] So far, however, no other studies analyzing the endocannabinoid system or the pharmacological effects of cannabinoids in human IBD have been published.

Gastrointestinal inflammation is likely the result of multiple factors, e.g., increased pro-inflammatory stimuli and reduced protective capability. The overall balance between pro- and anti-inflammatory mechanisms may determine the progression and severity of intestinal inflammation. [21], [22] Given the results of recent genome-wide association studies, [23] genetic susceptibility is an important factor contributing to IBD development. Moreover, knowledge of genetic susceptibility factors could provide important pathophysiologic insights for the generation of novel IBD therapeutics.

Considering our previous work on the endocannabinoid system in murine intestinal inflammation, [3], [6], [7], [24] we hypothesized that genetic variants in the *CNR1* gene, which may modulate CB_1_ receptor function, could be associated with an increased susceptibility to IBD. To test our hypothesis, we genotyped a cohort of more than 550 individuals including 382 IBD patients and analyzed whether the 1359 G/A (p.Thr453Thr; rs1049353) single nucleotide polymorphism (SNP) within the *CNR1* gene encoding the CB_1_ receptor modulates the susceptibility to CD and UC or results in a certain IBD phenotype. The selection of the *CNR1* 1359 G/A SNP was based on previous studies reporting that this polymorphism is associated with other disorders modulated by the endocannabinoid system such as alcohol dependence and hebephrenic schizophrenia. [25], [26]

## Methods

### Ethics Statement

The study was approved by the Ethics Committee of the Medical Faculty of the University of Munich. All participating subjects gave their written, informed consent prior to the genetic analysis.

### Human Study Population

The study population comprised 579 individuals, including 216 patients with CD, 166 with UC, and 197 healthy, unrelated controls. Patients and controls were recruited at the IBD center of the Ludwig-Maximilians-University Munich, Campus Grosshadern, from September 2002 to December 2006. The diagnoses of CD and UC were established following clinical guidelines, using endoscopic, radiological, and histopathologic criteria. Table 1 shows the baseline characteristics of the study population. All 197 controls were unrelated, healthy individuals of Caucasian origin and sex-matched (by frequency) to the CD group. Controls were healthy blood donors without a history or family history of IBD. Demographics and routine clinical data (including location and behavior of IBD, disease-related complications, and prescription data of immunosuppressive and immunomodulatory therapy e.g., azathioprine, 6-mercaptopurine, methotrexate, infliximab) were recorded by retrospective analysis of the clinical charts by two independent investigators and an interview including a questionnaire at the time of enrollment. All data were collected blind to the *CNR1* genotype. Patients with CD or UC were grouped according to age at diagnosis, disease localization, and behavior status of the Vienna classification, [27] and the recent modifications suggested by the Montreal classification. [28]

**Table 1:** Demographic characteristics of the study population.

	(1)CD (n = 216)	(2)UC (n = 166)	(3)Controls (n = 197)	(1) vs (2)p value	(1) vs (3)p value	(2) vs (3)p value
**Gender**						
Male (%)	105 (48.6%)	83 (50.0%)	114 (58.0%)	p = 0.84	p = 0.06	p = 0.14
Female (%)	111 (51.4%)	83 (50.0%)	83 (42.0%)			
**Age (yr)**						
Mean ± SD	41.4±11.8	43.3±14.4	43.9±21.6	p = 0.17	p = 0.19	p = 0.75
Range	17–71	19–85	0–80			
**Body mass index**						
Mean ± SD	23.1±3.9	24.2±4.2		p = 0.02		
Range	16–34	16–41				
**Age at diagnosis (yr)**						
Mean ± SD	28.1±11.4	31.8±13.7		p = 0.006		
Range	7–67	9–81				
**Disease duration (yr)**						
Mean ± SD	13.5±8.5	11.5±7.6		p = 0.016		
Range	2–44	1–40				
**Positive family history of IBD (%)**	30 (13.9%)	21 (12.7%)		p = 0.76		

### DNA Extraction and Genotyping of the CNR1 Polymorphism

Recently, a guanosine-to-adenine substitution at nucleotide position 1359 has been identified in the *CNR1* gene (rs1049353) [25], [29]. Thus, three genotypes (GG, GA, AA) are possible. Genomic DNA was isolated from peripheral blood leukocytes by standard procedures using the DNA blood mini kit from Qiagen (Hilden, Germany). Genotyping was done as previously described [7], [30]. Briefly, a single 20-µl PCR was performed to genotype this site using approximately 200 ng of genomic DNA and 20 pmol each of the following primers 5′-GAAAGCTGCATCAAGAGCCC-3′ (forward) and 5′-TTTTCCTGTGCTGCCAGGG-3′ (reverse). Other conditions were as follows: 1.5 mM MgCl_2_, 400 µM of each dNTP, 1.25 U Taq polymerase, and 1× reaction buffer (Life Technologies, Rockville, MD). DNA amplification was performed with 40 cycles of 94°C, 60°C, and 72°C for 30 seconds each, preceded by a single cycle of 95°C for 15 minutes and followed by a single cycle of 72°C for 5 minutes. Five µl of the resulting 111 bp PCR product were then digested overnight with 10 U of Mspl (New England Biolabs, Beverly, MA) at 37°C. This resulted in fragments of 92 and 19 bp, when a G was present at nucleotide position 385, while the fragment remained uncut, when an A was present. Restriction digests were analyzed by electrophoresis of the digestion mixture in a 2% agarose gel stained with ethidium bromide. The assay was verified by sequencing the PCR product and the digested PCR fragments of all possible genotypes.

### Statistics

Fisher's exact test was used for comparison between categorical variables. All tests were two-tailed. P values <0.05 were considered as significant. Analyses were performed using SPSS 14.0.1 software for Windows.

## Results

### The CNR1 1359 G/A (p.Thr453Thr) Polymorphism Modulates UC but Not CD Susceptibility

Given the above reported increased CB_1_ receptor expression in several models of intestinal inflammation and previous studies implicating the *CNR1* 1359 G/A (p.Thr453Thr) SNP in endocannabinoid-mediated diseases, [25], [31] we investigated whether this polymorphism modulates susceptibility and phenotype of CD and UC. The demographic characteristics of the IBD and control population analyzed are given in Table 1. The CD patients were classified with regard to their disease phenotype, considering disease location, the age at diagnosis, and disease behaviour by using the Montreal classification. [27], [28] The majority of patients had an onset of the disease in their mid20s (mean age at first diagnosis of CD: 28.1±11.4 years). The mean age at first diagnosis of UC was 31.8±13.7 years. 13.9% of the CD patients and 12.7% of the UC patients had a positive family history of IBD.

The results of the CNR1 p.Thr453Thr genotyping analysis in 216 CD patients, 166 UC patients, and 197 controls are shown in Table 2. The frequencies of heterozygous and homozygous carriers of this polymorphism did not differ significantly from the expected ratio according to the Hardy-Weinberg law. Patients with UC were less likely to be 1359 A/A homozygous (p = 0.01, OR 0.30, 95% CI 0.12–0.78). In contrast, this polymorphism did not influence susceptibility to CD (Table 2).

**Table 2:** Genotype frequencies of *CNR1* 1359 G/A (p.Thr453Thr) polymorphism in patients with Crohn's disease (CD) and ulcerative colitis (UC) as well as in controls.

	(1)GG	(2)GA	(3)AA	(1) vs. (2)	(1) vs. (3)
**CD**	115 (53.3%)	86 (39.8%)	15 (6.9%)	**CD vs. Controls**	
(n = 216)				p = 0.83	p = 0.28
**Controls** (n = 197)	103 (52.3%)	73 (37.0%)	21 (10.7%)	**UC vs. Controls**	
				p = 0.74	**p = 0.01OR 0.30CI 0.12–0.78**
**UC**	97 (58.4%)	63 (38.0%)	6 (3.6%)	**CD vs. UC**	
(n = 166)				p = 0.52	p = 0.17

### The CNR1 1359 G/A (p.Thr453Thr) Polymorphism Modulates Disease Onset and Body Mass Index in CD

In Table 3, we provide a detailed genotype-phenotype analysis of the *CNR1* 1359 G/A polymorphism in CD patients. Carriers of the 1359 A/A genotype were likely to have a lower body weight (p = 0.0005). In addition, homozygous carriers of the major G allele were more likely to develop CD before 40 years of age (p = 5.9×10^−7^) than carriers of the minor A allele. There was no association between the CNR1 p.Thr453Thr polymorphism and disease location, use of immunosuppressive drugs, family history of IBD, CD-related surgery, stenoses, and abscesses (Table 3). In addition, the CNR1 p.Thr453Thr polymorphism did not influence the UC phenotype (Table 4).

**Table 3:** Association between the *CNR1* 1359 G/A (p.Thr453Thr) genotype and CD characteristics.

	(1) GG(n = 114)	(2) GA(n = 86)	(3) AA(n = 15)	(1) vs (2)P value	(1) vs (3)P value	(1) vs (2)+(3)P value
**Male sex**	56 (49.1%)	41 (47.7%)	8 (53.3%)	p = 0.89	p = 0.79	p = 1.00
**Body mass index (kg/m** **^2^** **)**						
Mean ± SD	23.9±4.0	22.1±3.5	22.0±3.6	**p = 0.001**	**p = 0.05**	**p = 0.0005**
Range	16–34	16–32	18–31			
**Age at diagnosis (yr)**						
Mean ± SD	26.9±10.6	29.7±12.0	27.2±14.1	p = 0.09	p = 0.78	p = 0.11
Range	7–57	13–67	16–52			
**Disease duration (yr)**						
Mean ± SD	13.1±8.4	14.4±8.9	10.8±7.6	p = 0.31	p = 0.31	p = 0.52
Range	2–44	3–35	3–26			
**Age (yr)**						
Mean ± SD	39.9±11.5	44.0±11.7	37.2±12.1	**p = 0.01**	p = 0.46	**p = 0.05**
Range	17–70	23–71	19–56			
**Age at diagnosis**						
<17 years (A1)	0 (0.0%)	0 (0.0%)	0 (0.0%)	**p = 8.6×10^−8^**	p = 0.33	**p = 5.9×10^−7^**
17–40 years (A2)	89 (78.1%)	35 (40.7%)	10 (66.7%)			
>40 years (A3)	25 (21.9%)	51 (59.3%)	5 (33.3%)			
**Location**						
Terminal ileum (L1)	12 (10.5%)	8 (9.3%)	2 (13.3%)	p = 1.00	p = 0.67	p = 1.00
Colon (L2)	19 (16.7%)	18 (20.9%)	4 (26.7%)	p = 0.47	p = 0.47	p = 0.39
Ileocolon (L3)	60 (52.6%)	43 (50.0%)	7 (46.7%)	p = 0.78	p = 0.79	p = 0.68
Upper GI (L4)	23 (20.2%)	17 (19.8%)	2 (13.3%)	p = 1.00	p = 0.73	p = 0.86
Ileal involvement (L1 + L3)	72 (63.2%)	51 (59.3%)	9 (60.0%)	p = 0.66	p = 0.78	p = 0.18
**Behaviour**						
Non-sticturing, Non penetrating (B1)	22 (19.3%)	17 (19.8%)	4 (26.7%)	p = 1.00	p = 0.50	p = 0.86
Stricturing (B2)	33 (28.9%)	18 (20.9%)	3 (20.0%)	p = 0.25	p = 0.56	p = 0.21
Penetrating (B3)	59 (51.8%)	51 (59.3%)	8 (53.7%)	p = 0.32	p = 1.00	p = 0.34
**Use of immunosuppressive agents**	90 (78.9%)	64 (74.4%)	11 (73.3%)	p = 0.50	p = 0.74	p = 0.42
**Extraintestinal manifestations**	80 (70.2%)	55 (64.0%)	11 (73.3%)	p = 0.36	p = 1.00	p = 0.47
**Positive family history of IBD**	29 (25.4%)	13 (15.1%)	3 (20.0%)	p = 0.08	p = 0.76	p = 0.09
**Surgery because of CD**	65 (57.0%)	54 (62.8%)	8 (53.3%)	p = 0.47	p = 0.79	p = 0.58
**Fistulas**	59 (51.8%)	51 (59.3%)	8 (53.3%)	p = 0.32	p = 1.00	p = 0.34
**Stenosis**	78 (68.4%)	58 (67.4%)	9 (60.0%)	p = 0.88	p = 0.56	p = 0.77
**Abscesses**	41 (36.0%)	32 (37.2%)	4 (26.7%)	p = 0.88	p = 0.57	p = 1.00

1 Disease behavior was defined according to the Montreal classification [28]. A stricturing disease phenotype was defined as presence of stenosis without penetrating disease. The diagnosis of stenosis was made surgically, endoscopically, or radiologically (using MRI enteroclysis).

2 Immunosuppressive agents included azathioprine, 6-mercaptopurine, 6-thioguanin, methotrexate, and/or infliximab.

3 Extraintestinal manifestations were defined as one or more of the following IBD-related diseases: non-medication-induced arthropathies (e.g., ankylosing spondylitis, sacroileitis, peripheral arthritis), eye involvement (e.g., episcleritis and/or iritis/uveitis), skin involvement (e.g., erythema nodosum and pyoderma gangrenosum), non-medication-induced biliary disease (e.g., sclerosing cholangitis).

4 Only surgery related to CD-specific problems (e.g., fistulectomy, colectomy, ileostomy) was included.

**Table 4:** Association between *CNR1* 1359 G/A (p.Thr453Thr) genotype and UC disease characteristics.

	(1)GG(n = 97)	(2)GA(n = 63)	(3)AA(n = 6)	(1) vs. (2)p value	(1) vs. (3)p value	(2) vs. (3)p value	(1) vs. (2)+(3)p value
**Male sex**	47 (48.5%)	32 (50.8%)	4 (66.7%)	p = 0.87	p = 0.44	p = 0.68	p = 0.75
**Body mass index (kg/m** **^2^** **)**							
Mean ± SD	23.8±3.7	24.4±4.9	26.5±4.6	p = 0.46	p = 0.27	p = 0.39	p = 0.30
Range	16–32	18–41	20–32				
**Age at diagnosis (yr)**							
Mean ± SD	31.7±13.9	31.7±13.4	34.5±17.0	p = 0.99	p = 0.70	p = 0.70	p = 0.92
Range	9–73	14–81	13–57				
**Disease duration (yr)**							
Mean ± SD	11.9±6.5	12.3±8.7	14.2±11.1	p = 0.79	p = 0.64	p = 0.70	p = 0.68
Range	2–2 9	2–41	4–36				
**Age (yr)**							
Mean ± SD	43.7±14.2	43.9±15.1	48.7±11.9	p = 0.93	p = 0.37	p = 0.40	p = 0.79
Range	21–81	20–86	37–68				
**Location**							
Rectum	21 (21.7%)	7 (11.1%)	1 (16.7%)	p = 0.13	p = 1.00	p = 0.55	p = 0.10
Left-sided	28 (28.9%)	28 (44.4%)	2 (33.3%)	p = 0.06	p = 1.00	p = 0.69	p = 0.07
Pancolitis	48 (49.4%)	28 (44.4%)	3 (50.0%)	p = 0.63	p = 1.00	p = 1.00	p = 0.64
**Use of immunosuppressive agents**	67 (69.1%)	48 (76.2%)	4 (66.7%)	p = 0.37	p = 1.00	p = 0.63	p = 0.39
**Use of infliximab**	27 (27.8%)	11 (17.5%)	1 (16.7%)	p = 0.18	p = 1.00	p = 1.00	p = 0.14
**Surgery due to UC**	4 (4.1%)	0 (0.0%)	0 (0.0%)	p = 0.15	p = 1.00	p = 1.00	p = 0.14
**Fistulas**	4 (4.1%)	3 (4.8%)	0 (0.0%)	p = 1.00	p = 1.00	p = 1.00	p = 1.00
**Stenosis**	15 (15.5%)	4 (6.3%)	1 (16.7%)	p = 0.13	p = 1.00	p = 0.37	p = 0.15
**Abscesses**	5 (5.2%)	4 (6.3%)	0 (0.0%)	p = 0.74	p = 1.00	p = 1.00	p = 1.00
**Extraintestinal manifestations**	14 (14.4%)	11 (17.5%)	0 (0.0%)	p = 0.66	p = 1.00	p = 0.58	p = 0.83
**Positive family history**	15 (15.5%)	6 (9.5%)	0 (0.0%)	p = 0.34	p = 0.59	p = 1.00	p = 0.24

1 Immunosuppressive agents included azathioprine, 6-mercaptopurine, and/or infliximab.

2 Only UC-related surgery (e.g., colectomy) was included.

3 Extraintestinal manifestations were defined as one or more of the following IBD-related diseases: non-medication-induced arthropathies (e.g., ankylosing spondylitis, sacroileitis, peripheral arthritis), eye involvement (e.g., episcleritis and iritis/uveitis), skin involvement (e.g., erythema nodosum and pyoderma gangrenosum), non-medication-induced biliary disease (e.g., sclerosing cholangitis).

## Discussion

We analyzed the effect of the CNR1 p.Thr453Thr polymorphism on IBD susceptibility and disease phenotype. This study was based on our previous results which suggested that CB_1_ receptor signaling is involved in defense mechanisms in response to acute intestinal inflammation in animal models. [3], [7] Based on this knowledge, we hypothesized that differential CB_1_ receptor expression, e.g., modulated by genetic factors, may contribute to IBD susceptibility. In the present study, we focused on the 1359 G/A polymorphism within the *CNR1* gene encoding the CB_1_ receptor, given the importance of this nucleotide substitution in other endocannabinoid-mediated disorders such as alcohol dependence [25] and schizophrenia [32]. Although the CNR1 1359 G/A (p.Thr453Thr) SNP is a silent mutation, which does not result in an amino acid exchange, it might be associated with alterations e.g. in RNA splicing. [33]

Our study demonstrated an association with UC susceptibility but not with CD susceptibility. The prevalence of 1359 A/A homozygous carriers was 10.7% in the control population, 6.9% in CD patients, and only 3.6% in UC patients. The genotype frequencies found for our control population were within the values expected from Hardy-Weinberg equilibrium and were similar to a previous German control cohort. [29] However, given the limited size of the study population and the low prevalence of 1359 A/A homozygous carriers among UC patients, this finding has to be confirmed in larger replication cohorts. Furthermore, given that the study was primarily designed to detect differences in the frequency of IBD risk alleles, the control population was selected only regarding absence of IBD and other chronic diseases as well as being negative for a family history of IBD. Therefore a selection bias resulting in differences e.g. in BMI can not be excluded though it is intriguing that a lower BMI was found with CD and not with UC.

The human *CNR1* gene is localized on chromosome 6q14–q15. [34] Interestingly, an earlier genome-wide family-based linkage study found an association of this region with celiac disease [35]. We recently demonstrated that celiac disease and UC (but not CD) share another common susceptibility locus on chromosome 4q27. [36] Although none of the recent genome-wide association studies demonstrated the *CNR1* gene as a major IBD susceptibility gene, a previous genome scan in 260 IBD-affected relative pairs found nominal evidence for linkage of IBD to loci on chromosome 6q (lod = 2.21 between D6S2436/D6S305). [37]

We recently confirmed a number of CD susceptibility genes found in genome-wide associations studies such as *NOD2*, [38], [39] *IL23R*, [40] and *ATG16L1* [41], but we also demonstrated differences in the genetic susceptibility to CD [42], suggesting that there are differences in the genetic susceptibility to IBD even between different Caucasian populations. In addition, other genetic associations such as those of *TLR4* SNPs with CD susceptibility shown by our group [43] were not among the major CD susceptibility genes in a recent meta-analysis of genome-wide scans, although this gene has been confirmed as a CD susceptibility gene. [44]

Currently, it is unknown if the *CNR1* 1359 G/A (p.Thr453Thr) SNP modulates CB_1_ receptor expression or function. Particularly changes at amino acid positions 418–439 seem to be associated with a lack of receptor desensitization [45], and allelic variation in the *CNR1* gene was suggested to be associated with a lower rather than a higher receptor activity, [46] but detailed studies investigating receptor activity based on different *CNR1* genotypes are lacking. The assumption that allelic variation is associated with reduced CB1 activity would also explain the low BMI found in the 1359 A/A homozygous CD patients. Consistent with these data, the 1359 G/G wildtype genotype has been shown to be associated with an increased BMI and overweight in an Italian study of a healthy population. [47] A low BMI in CD patients is also considered to be an indicator of high disease activity. Therefore, 1359 A/A homozygosity could contribute to a more severe disease phenotype. This would be consistent with our results in *CNR1^−/−^* mice, demonstrating that these knockout mice take a more fulminate course in DNBS and DSS colitis. [3] However, functional experiments have to analyze if CNR1 signaling is indeed decreased in 1359 A/A homozygous patients.

Our findings add evidence that targeting the CB_1_ receptor system may modulate intestinal inflammation, suggesting this receptor as a potential target for future treatments. Similarly, animal models suggest that CB_1_ receptor activation with exogenous CB_1_ receptor agonists induces protection against intestinal inflammation. [3], [5] Therefore, the increased CB_1_ receptor expression seen in murine colitis models is likely an intrinsic protective mechanism to counter-regulate the deleterious effects of intestinal inflammation. The physiological importance of the CB_1_ receptor and the endocannabinoid system becomes obvious when endocannabinoid levels are increased by blocking their degradation. Under these circumstances, intestinal inflammation is reduced and the CB_1_ receptor is involved in this protection, emphasizing the important pathophysiological role of this system in intestinal inflammation. [7] Whether monitoring of CB_1_ receptor function or genotyping can identify responders of future treatments targeting the CB_1_ receptor remains speculative and has to be clarified in clinical trials.

In summary, we demonstrate that the *CNR1* 1359 G/A polymorphism modulates IBD susceptibility and phenotype. Specifically, we show that 1359 A/A homozygosity protects against UC and that CD patients carrying the minor A allele have a later disease onset and a lower BMI. These findings have to be confirmed in a larger replication study. Given the low prevalence of 1359 A/A homozygous carriers, this likely can be achieved only in a large multicenter trial. Nevertheless, our findings provide further evidence that endocannabinoids modulate intestinal inflammation, suggesting that this system could act as a target for future therapeutic interventions.
